# The Reading Everyday Emotion Database (REED): a set of audio-visual recordings of emotions in music and language

**DOI:** 10.1007/s10579-023-09698-5

**Published:** 2023-11-20

**Authors:** Jia Hoong Ong, Florence Yik Nam Leung, Fang Liu

**Affiliations:** 1 School of Psychology and Clinical Language Sciences, University of Reading, Harry Pitt Building, Earley Gate, Reading, RG6 6AL UK; 2 Department of Psychology, School of Social Sciences, Nottingham Trent University, Nottingham, UK; 3 Department of Psychology, University of Bath, Bath, UK

**Keywords:** Emotion, Database, Audio-visual, Stimulus set, Speech, Song

## Abstract

Most audio-visual (AV) emotion databases consist of clips that do not reflect real-life emotion processing (e.g., professional actors in bright studio-like environment), contain only spoken clips, and none have sung clips that express complex emotions. Here, we introduce a new AV database, the Reading Everyday Emotion Database (REED), which directly addresses those gaps. We recorded the faces of everyday adults with a diverse range of acting experience expressing 13 emotions—neutral, the six basic emotions (angry, disgusted, fearful, happy, sad, surprised), and six complex emotions (embarrassed, hopeful, jealous, proud, sarcastic, stressed)—in two auditory domains (spoken and sung) using everyday recording devices (e.g., laptops, mobile phones, etc.). The recordings were validated by an independent group of raters. We found that: intensity ratings of the recordings were positively associated with recognition accuracy; and the basic emotions, as well as the Neutral and Sarcastic emotions, were recognised more accurately than the other complex emotions. Emotion recognition accuracy also differed by utterance. Exploratory analysis revealed that recordings of those with drama experience were better recognised than those without. Overall, this database will benefit those who need AV clips with natural variations in both emotion expressions and recording environment.

**Supplementary Information:**

The online version contains supplementary material available at 10.1007/s10579-023-09698-5.

## Introduction

Affective science is an interdisciplinary field of research that examines research questions related to emotion. To address some of those questions, various emotional stimuli corpora or databases have been developed (for a review of some of these databases, see Krumhuber et al., 2017; Wu et al., 2014). For example, psychologists may develop a stimulus set for experimental studies on emotion perception (e.g., Benda & Scherf, 2020; Thompson et al., 2013) and computer scientists may create a corpus of recordings to train machine learning models to annotate emotions automatically (e.g., Cosker et al., 2011; Yin et al., 2008). The development of these databases is often time-consuming and resource-intensive, but fortunately, most of these databases are made available and shared with other researchers. Indeed, in recent years, there has been great progress made in the machine learning field to classify emotions using audio-visual stimuli from existing databases (e.g., Ma et al., 2019; Praveen et al., 2022; Schoneveld et al., 2021). This paper describes the development of one such audio-visual (AV) database that complements the existing ones in the field: the Reading Everyday Emotion Database (REED).^1^

Most previous databases tend to be unimodal, that is, the stimuli are either auditory-only (AO) or visual-only (VO). Some examples of the AO databases are the Macquarie Battery of Emotional Prosody (Thompson et al., 2013), the EU-Emotion Voice Database (Lassalle et al., 2019), and the Vocal Expressions of Nineteen Emotions across Cultures (VENEC) corpus (Laukka et al., 2010). These AO databases have verbal vocalisations (e.g., spoken utterances in particular emotions) and/or non-verbal vocalisations such as laughs or screams. The VO databases contain stimuli that are either static (i.e., still photographs or images)—such as the NimStim database (Tottenham et al., 2009) and the Facial Expression of Emotion—Stimuli and Tests (FEEST) (Young et al., 2002), which uses photographs from the classical set Picture of Facial Affect (Ekman & Friesen, 1976)—or dynamic (i.e., silent videos) created using morphs of still images (e.g., from neutral expression to angry) (Montagne et al., 2007; Young et al., 2002) or video-recordings presented without the audio (Golan et al., 2006; O’Toole et al., 2005; van der Schalk et al., 2011; Wingenbach et al., 2016).

Stimuli from these databases have often been used to investigate emotion perception in various studies, and by far, the most used ones are the static VO databases (i.e., the still photographs). Some have criticised the use of still photographs to investigate emotion perception, since the temporal, dynamic information of emotions is crucial for emotion processing (Krumhuber et al., 2013) and human perceivers tend to integrate both auditory (e.g., acoustic) and visual (e.g., facial) cues for emotion recognition (Massaro & Egan, 1996). Indeed, direct comparisons of unimodal (AO or VO) vs. bimodal (AV) presentations of emotions revealed that human perceivers are more accurate at recognising emotions (Kim & Davis, 2012) and rate emotions as more intense (Bhullar, 2013) when presented in AV mode. Thus, to increase ecological validity of emotion perception research (and affective science, generally), AV databases are needed.

There are two main types of AV databases in the field: those that involve naturalistic or interaction-based recordings and those that involve posed recordings. The former typically consists of clips from television shows/films as stimuli in the database (Dhall et al., 2012; Douglas-Cowie et al., 2011), recordings of spontaneous reactions of individuals watching clips (Ringeval et al., 2013), or recordings of one or more individuals interacting or performing a task (Busso et al., 2008). Recordings from these databases often have situational cues to aid emotion expression and the verbal content may not be the same across actors, which, though useful for those investigating spontaneous and naturalistic emotions, may pose a challenge for those who need precise control over the stimuli. The posed AV databases offer such control given that the actors typically use the same set of contents or utterances to produce the same set of emotions. Table 1 presents some examples of posed AV databases in the field. These posed AV databases nonetheless have certain limitations: most consist only of a small range of emotions (typically, the six ‘basic’ emotions—angry, disgusted, fearful, happy, sad, and surprised —and neutral), and are recorded by professional actors and thus may display exaggerated expressions (Jürgens et al., 2015). Moreover, all the databases were recorded in pristine, studio-like conditions with bright lighting, plain-coloured background, high-definition camera, and clear audio. In other words, the currently available posed AV databases may not reflect how emotions are expressed in a typical, ‘real world’ setting (e.g., during teleconferencing) where not only do the expressers may not have any acting experience, but the recording conditions may also differ variably (e.g., the lighting level and colour saturation between clips may vary naturally between clips compared to that of studio recordings).

**Table 1:** List of posed audio-visual databases and information on their recordings including the location (Location); whether they were speech, song, or both (Domain); the language of the recording (Language); the number of encoders (No. Encoder) and whether they were professional/experienced actors (Pro?); and the list of emotions recorded

Name	Location	Domain	Language	No. Encoder	Pro?	Emotions
Audio-visual database of emotional speech in Basque (Navas et al., 2004)	Studio/Lab	Speech	Basque	1	Yes	Angry, Disgust, Fearful, Happy, Neutral, Sad, Surprise
Database of Kinetic Facial Expressions (DaFEx) (Battocchi et al., 2005)	Studio/Lab	Speech	Italian	8	Yes	Angry, Disgust, Fearful, Happy, Neutral, Sad, Surprise
Geneva Multimodal Emotion Portrayals—Core Set (GEMEP-CS) (Bänziger et al., 2012)	Studio/Lab	Speech	Pseudospeech	10	Yes	Admiration, Amusement, Anxiety, Contempt, Cold anger, Despair, Disgust, Hot anger, Fear, Interest, Joy, Pleasure, Pride, Relief, Sadness, Surprise, Tenderness
Multimedia Human–Machine Communication (MHMC) Database (Lin et al., 2012)	Studio/Lab	Speech	Chinese	7	No	Angry, Happy, Neutral, Sad
Surrey Audio-Visual Expressed Emotion (SAVEE) Database (Haq & Jackson, 2009)	Studio/Lab	Speech	English (British)	4	No	Angry, Disgust, Fearful, Happy, Neutral, Sad, Surprise
The EU-Emotion Stimulus Set (O’Reilly et al., 2016)	Studio/Lab	Speech	English (British)	19	Yes	Afraid, Angry, Ashamed, Bored, Disappointed, Disgusted, Excited, Frustrated, Happy, Hurt, Interested, Jealous, Joking, Kind, Neutral, Proud, Sad, Sneaky, Surprise, Unfriendly, Worried
The Ryerson Audio-Visual Database of Emotional Speech and Song (RAVDESS) (Livingstone & Russo, 2018)	Studio/Lab	Speech & Song	English (Canadian)	24	Yes	Speech: Angry, Calm, Disgust, Fearful, Happy, Neutral, Sad, Surprise; Song: Angry, Calm, Fearful, Happy, Neutral, Sad
The STOIC Dynamic Facial Emotional Expressions Database (Roy et al., 2007)	Studio/Lab	Speech	French (Montreal)	34	Yes	Angry, Disgust, Fearful, Happy, Neutral, Pain, Sad, Surprise

As can be seen in Table 1, there is a paucity of databases that include sung emotions, which is regrettable as this presents a barrier to cross-domain emotion research. Indeed, given that speech and song are human-specific vocal channels, there is a lot of interest in studying the similarities and differences between the two. Yet relatively little is known about their similarities and differences in emotion expression, presumably, partly due to the lack of resources available. Understanding how the two domains are related in their emotion expression will not only deepen our understanding of the potential shared mechanism between them, but may also have implications for the development of emotion skill interventions such as for individuals with autism or alexithymia (Allen & Heaton, 2010; Katagiri, 2009). In the one database that does include sung emotions (RAVDESS), only six emotions were examined (angry, calm, fearful, happy, neutral, and sad), which limits the generalisability of comparative studies between speech and song to other (complex) emotions.

We developed the REED to complement the existing posed AV databases by addressing those limitations. The recordings from the REED are devoid of situational cues, similar to the previous posed AV databases. However, unlike the previous ones, we set out to record a wider range of emotions (neutral, the six basic emotions, and six complex emotions—embarrassed, hopeful, jealous, proud, sarcastic, and stressed) with adults across ages with and without acting/drama experience (the ‘encoders’) to better reflect the general population who may have varying levels of acting experience. We also aimed to expand the available AV databases by including both speech and song domains, the latter of which is scarcely available in the field, and thus enable comparative studies in spoken vs. sung emotions that are not limited to basic emotions. To ensure variability in the recording conditions, we recorded encoders using everyday recording devices commonly used in teleconferencing (i.e., their own webcam, mobile phone, etc.).^2^

## Methods

### Stimulus creation

#### Participants

Twenty-two adults (12 females and 10 males; hereafter ‘encoders’) participated in the stimulus creation phase, representing a diverse age range (19–81 years old, Mean = 38.18, SD = 19.49, *n* 18–40 years old = 15, *n* 41 + years old = 7). The encoders were all native British English speakers, and none reported having any neurological impairments or speech, hearing, or visual difficulties. Approximately half (*n* = 12) reported having some musical training experience,^3^ among which their cumulative musical experience summing across multiple instruments, if any, spanned between 5 and 52 years (Mean = 13.75, SD = 13.53). Only one considered themselves a professional musician and five no longer practise music. Nine of the encoders reported having some drama experience—with four considering themselves professionals (2 actors, 1 actor/director, and 1 drama teacher), and the other five had amateur drama experience. See Supplementary Section S1 for a detailed description of each encoder and the device they used for recording. The encoders were briefed of the nature of the study and gave their written informed consent prior to their participation in the stimulus creation task. They were given monetary compensation or course credit for their time. The study protocol was reviewed and approved by the University Research Ethics Committee (UREC) at the University of Reading.

#### Task & procedure

The stimulus creation phase was conducted virtually on Microsoft Teams, and the encoders used their own devices (e.g., webcam, mobile phone, etc.) for the recording. The encoders completed four conditions: three spoken utterances of various lengths (“Ah”, “Happy birthday to you”, and “The music played on while they talked”) and one sung utterance (singing the first line of “Happy birthday to you”). The different lengths of the spoken utterances from the single-syllable “Ah”^4^ to the sentence-long “The music played on while they talked” were chosen to enable the REED users more options that would better fit their research questions and to enable the examination of stimulus duration as a factor in their research. We chose the utterance “Happy Birthday to you” to be both spoken and sung by the encoders to enable the REED users to compare speech vs. song emotion processing directly, given that they have the same verbal content and that it is easily recognised by most. On a more practical note, we asked the encoders to sing “Happy birthday to you” because we believe it may be more accessible for the encoders to sing due to its familiarity, particularly those who are not musically trained. If we were to use a novel melody, participants will not only have to learn the new melody, but also be recorded singing it and expressing emotions, which may be intimidating for the non-musically trained participants. Half the encoders recorded the spoken utterances first and the rest did the sung utterance first. Within the spoken domain, the order of the conditions was completely randomised for each encoder. For the sung condition, the encoders sang only the first line of “Happy birthday to you” that is, the first six notes of the song. The encoders were asked to sing within their comfortable octave range, and they were not required to pitch- or rhythm-match to a standard melody; the only requirement was that the melody should be recognisable to the experimenters as the first line of “Happy birthday to you”. Within each condition, they produced the utterance in 13 different emotions: first in neutral, then followed by (in random order): angry, disgusted, embarrassed, fearful, happy, hopeful, jealous, proud, sad, sarcastic, stressed, and surprised. The encoders were given the definition and a scenario^5^ for each emotion (see Supplementary Section S2) prior to the recording, and they were instructed to use the scenario to help them elicit the emotion. The encoders recorded at least five takes of each emotion for each condition; thus, the encoders recorded 260 trials at a minimum (4 conditions × 13 emotions × 5 takes).

The recording was conducted with each encoder individually via Microsoft Teams. Prior to the recording, the experimenters (JHO and FYNL) made sure the encoder’s background was as plain as possible and the lighting was sufficient that the encoder could be seen (though note that the recording conditions may differ across all the encoders —see Supplementary Section S3—and it is this variation in the recording conditions that we believe is one of the unique features of the REED). In addition to recording the session on Microsoft Teams, one of the experimenters (JHO) also recorded their screen as a previous version of Microsoft Teams recorded windows from all the attendees of the online meeting, thus making the encoder’s screen appear smaller in the recorded sessions. During the recording, the other experimenter (FYNL) prompted the encoder and asked for retakes should there have been any disruptions (e.g., background noise, connection issues, video lag, etc.). Importantly, unlike some (e.g., EU-Emotion) but not all of the existing databases (e.g., RAVDESS), neither of the experimenters coached the encoder on how to produce the emotions, thus allowing for natural variation in emotion expression to occur across different encoders. The recording was self-paced, and the encoder was encouraged to take a short break whenever necessary. The recording took approximately one hour to complete.

#### Post-recording processing

**Fig. 1:**
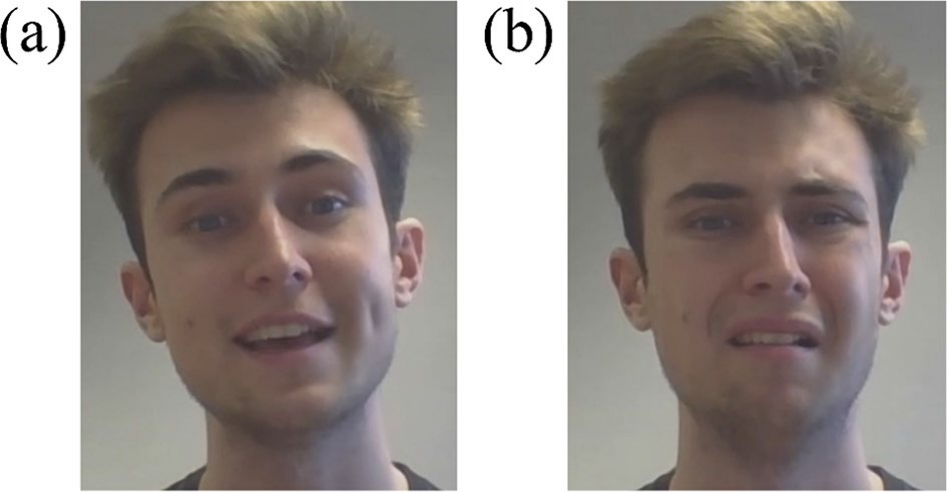
Screenshots of an encoder depicting **a** happy, and **b** disgust

For each encoder, we first synchronised the video from the screen-recording and the audio from the recording on Microsoft Teams using DaVinci Resolve (2019). The synchronised video was then exported as an audio-visual file of mp4 format with 24 frames-per-second (fps) and a resolution of 1920 × 1080 pixels. Next, the audio-visual file was segmented into individual clips, each of which depicts an emotion for each condition, using DaVinci Resolve. The segmented clips were cropped to display just the face region of the encoder, and the cropped clips were then resized to the resolution of 396 × 512 pixels, to ensure a standardised resolution across all the clips.^6^ See Fig. 1 for screenshots of two clips.

The two experimenters (JHO and FYNL) rated each clip on its genuineness (i.e., how believable the emotion expression was), quality (i.e., how good the overall quality was in terms of its usability as an experimental stimulus—e.g., whether there was background noise, lag in the video, how good an exemplar the sung melody was to the canonical “Happy birthday” song, etc.), valence, and arousal on a 5-point scale. Based on the genuineness and quality ratings, we selected up to the best three tokens for each emotion per condition and encoder to be validated (see ‘Stimulus Validation’ subsection below).

### Stimulus validation

#### Participants

The participants for the stimulus validation phase consisted of 168 adults (90 females and 78 males; hereafter ‘decoders’) whose age ranged between 18 and 45 (Mean = 33.38, SD = 7.40). Twenty-two participants were additionally tested but were excluded as they did not meet the threshold for their performances on the catch trials (see ‘Task & Procedure’ subsection below). All but one of the decoders were native British English speakers (*n* = 167), and of the one who was not, they self-rated their English proficiency to be 6 on a 7-point scale with 7 being native-like. None of the decoders reported having neurological impairments or speech, hearing, or visual difficulties. About a quarter of the decoders (*n* = 43) reported having some musical training experience (cumulative experience: Mean = 9.93 years, SD = 13.12, Range = 1–66) and considerably less decoders (*n* = 19) reported having some amateur drama experience (e.g., being in youth theatre, studied drama in school, etc.; Mean = 5.13 years, SD = 5.37, range = 0.5–23). The decoders gave their written informed consent prior to their participation, and they received monetary compensation for their time. The study protocol was reviewed and approved by the University Research Ethics Committee (UREC) at the University of Reading.

#### Task & procedure

Stimulus validation was done using a forced-choice recognition task in which participants were presented with all the possible labels, similar to previous studies (e.g., Bänziger et al., 2012). The task was conducted online on the Gorilla Experiment Builder (Anwyl-Irvine et al., 2020). There were 3230 clips to be validated in total (22 encoders × 13 emotions × 4 conditions × between 1 and 3 clips per encoder), which were divided into 15 lists, with each list having either 225 or 226 clips. At least 11 decoders were randomly assigned to each list; thus, each clip had at least 11 responses, similar to a previous study (Livingstone & Russo, 2018). On each trial, the decoders were presented with a clip, followed by all 13 labels. Two sets of button orders were generated, and participants were randomly allocated to a set at the start of the task. After each recognition response, the decoders then rated each clip for their intensity and genuineness on a 5-point scale. To ensure attentiveness, 10 catch trials, which consisted of a grey-scale clip or a clip with an audio beep, were presented randomly and participants were instructed to select the last button in each set for those catch trials. Participants were removed from the analysis if they scored less than 50% accuracy on the catch trials. Participants were given an opportunity for a short break after every 25 trials and the task took approximately an hour to complete.

### Data analysis

We examined the influence of intensity, genuineness, emotion, and condition on the recognition accuracy of the intended emotions in the clips in the database by fitting a binomial generalised linear mixed effects model using the *lme4* package (Bates et al., 2015) in R (R Core Team, 2021), with the binary variable Accuracy (Correct/Incorrect) as the dependent variable. As predictor variables, we entered Intensity and Genuineness (both mean-centred continuous variables), as well as Emotion and Condition (both categorical variables) and the interaction between the two categorical variables. We tried modelling the random effects as maximal as possible (Barr et al., 2013), but due to convergence issues, we only included by-subject and by-item intercepts and by-subject slopes for Emotion and for Condition as random effects in the model.

Though the study was not designed to address individual differences and recognition performance, we also explored whether musical and drama experience of the encoders and decoders may influence recognition performance as a function of Condition. To that end, we fitted another binomial generalised linear mixed effects model with Accuracy (Correct/Incorrect) as the dependent variable, and Encoder Musicianship (Nonmusician vs. Musician), Encoder Drama Experience (Without vs. With), Decoder Musicianship (Nonmusician vs. Musician), Decoder Drama Experience (Without vs. With), Condition, and all the two-way interactions involving Condition and the Musical/Drama experience as fixed effects. By-subject and by-item random intercepts and random slopes for Condition were also included as random effects.

For both models, marginal R2 and conditional R2 values, which reflect the variance explained only by fixed effects and by both the fixed effects and random effects, respectively, were estimated using the *rsquared.glmm()* function (Lefcheck & Casallas, 2014). Statistical significance of each predictor was determined using the function *Anova*() from the *car* package (Fox & Weisberg, 2019). Subsequent pairwise comparisons with Tukey corrections were conducted using the *emmeans* package (Lenth, 2019).

### Results

Table 2 summarises the mean recognition performance and the mean intensity and genuineness ratings for each emotion overall (collapsing across the four conditions) and by condition. As seen in Table 2, the basic emotions (Angry, Disgusted, Fearful, Happy, Sad, and Surprised), Neutral, and Sarcastic emotions were generally recognised better than the other complex emotions (Embarrassed, Hopeful, Jealous, Proud, and Stressed).

**Table 2:** Mean recognition accuracy, and mean ratings of intensity and genuineness (standard deviations in parentheses) for each emotion overall (collapsing across conditions), and for each emotion by condition

Emotion	Mean accuracy (SD)	Mean intensity (SD)	Mean genuineness (SD)
Overall			
Angry	0.45 (0.50)	3.49 (1.06)	3.17 (1.17)
Disgusted	0.46 (0.50)	3.47 (1.05)	3.12 (1.20)
Embarrassed	0.12 (0.33)	3.11 (1.02)	3.10 (1.12)
Fearful	0.34 (0.47)	3.28 (1.06)	3.06 (1.16)
Happy	0.49 (0.50)	3.51 (1.07)	3.42 (1.18)
Hopeful	0.11 (0.31)	3.23 (1.04)	3.20 (1.14)
Jealous	0.11 (0.31)	3.24 (1.09)	3.18 (1.15)
Neutral	0.55 (0.50)	3.05 (1.13)	3.24 (1.11)
Proud	0.11 (0.32)	3.32 (1.05)	3.29 (1.15)
Sad	0.50 (0.50)	3.24 (1.09)	3.13 (1.15)
Sarcastic	0.34 (0.47)	3.27 (1.11)	3.15 (1.16)
Stressed	0.17 (0.38)	3.23 (1.03)	3.12 (1.12)
Surprised	0.50 (0.50)	3.49 (1.04)	3.21 (1.19)
Speech “Ah”			
Angry	0.43 (0.50)	3.31 (1.07)	2.95 (1.19)
Disgusted	0.53 (0.50)	3.42 (1.06)	3.19 (1.20)
Embarrassed	0.15 (0.36)	3.01 (1.04)	3.10 (1.15)
Fearful	0.29 (0.45)	3.15 (1.12)	2.94 (1.23)
Happy	0.27 (0.45)	3.35 (1.15)	3.26 (1.25)
Hopeful	0.10 (0.30)	3.12 (1.10)	3.02 (1.24)
Jealous	0.03 (0.18)	2.93 (1.10)	3.04 (1.15)
Neutral	0.42 (0.49)	2.78 (1.18)	2.99 (1.18)
Proud	0.07 (0.26)	3.08 (1.07)	3.10 (1.18)
Sad	0.41 (0.49)	2.96 (1.10)	3.05 (1.20)
Sarcastic	0.31 (0.46)	3.08 (1.13)	2.97 (1.20)
Stressed	0.22 (0.41)	3.12 (1.04)	3.02 (1.21)
Surprised	0.79 (0.41)	3.52 (1.08)	3.23 (1.28)
Speech “Talked”			
Angry	0.47 (0.50)	3.52 (1.06)	3.34 (1.11)
Disgusted	0.46 (0.50)	3.49 (1.08)	3.17 (1.15)
Embarrassed	0.11 (0.31)	3.11 (1.02)	3.16 (1.10)
Fearful	0.35 (0.48)	3.38 (1.04)	3.08 (1.11)
Happy	0.44 (0.50)	3.50 (1.05)	3.37 (1.13)
Hopeful	0.13 (0.34)	3.28 (1.03)	3.33 (1.07)
Jealous	0.07 (0.26)	3.23 (1.06)	3.26 (1.12)
Neutral	0.68 (0.47)	3.25 (1.09)	3.49 (1.08)
Proud	0.16 (0.37)	3.36 (1.07)	3.36 (1.10)
Sad	0.55 (0.50)	3.39 (1.09)	3.30 (1.11)
Sarcastic	0.21 (0.41)	3.22 (1.05)	3.24 (1.10)
Stressed	0.22 (0.42)	3.35 (1.07)	3.30 (1.11)
Surprised	0.57 (0.50)	3.58 (1.00)	3.32 (1.16)
Speech “Birthday”			
Angry	0.51 (0.50)	3.68 (1.06)	3.33 (1.18)
Disgusted	0.45 (0.50)	3.60 (1.03)	3.10 (1.24)
Embarrassed	0.10 (0.30)	3.18 (0.98)	3.05 (1.10)
Fearful	0.32 (0.47)	3.29 (1.02)	3.09 (1.15)
Happy	0.60 (0.49)	3.59 (1.00)	3.55 (1.17)
Hopeful	0.12 (0.32)	3.22 (1.01)	3.15 (1.12)
Jealous	0.18 (0.39)	3.47 (1.05)	3.29 (1.15)
Neutral	0.58 (0.49)	3.12 (1.09)	3.30 (1.05)
Proud	0.13 (0.33)	3.46 (0.98)	3.34 (1.14)
Sad	0.51 (0.50)	3.28 (1.05)	3.10 (1.14)
Sarcastic	0.46 (0.50)	3.49 (1.13)	3.20 (1.19)
Stressed	0.14 (0.35)	3.28 (1.01)	3.09 (1.07)
Surprised	0.42 (0.49)	3.54 (1.04)	3.22 (1.19)
Song “Birthday”			
Angry	0.41 (0.49)	3.43 (1.02)	3.06 (1.16)
Disgusted	0.36 (0.48)	3.37 (1.03)	3.00 (1.19)
Embarrassed	0.12 (0.33)	3.15 (1.04)	3.10 (1.10)
Fearful	0.40 (0.49)	3.30 (1.05)	3.15 (1.12)
Happy	0.69 (0.46)	3.64 (1.05)	3.56 (1.15)
Hopeful	0.08 (0.27)	3.29 (0.99)	3.31 (1.11)
Jealous	0.15 (0.35)	3.34 (1.08)	3.13 (1.17)
Neutral	0.53 (0.50)	3.09 (1.09)	3.21 (1.07)
Proud	0.09 (0.28)	3.38 (1.04)	3.37 (1.15)
Sad	0.55 (0.50)	3.35 (1.08)	3.10 (1.15)
Sarcastic	0.38 (0.48)	3.31 (1.07)	3.18 (1.12)
Stressed	0.10 (0.30)	3.16 (0.99)	3.07 (1.04)
Surprised	0.18 (0.38)	3.27 (1.03)	3.04 (1.12)

*Speech “Ah”* “Ah” spoken condition,* Speech “Talked”* “The music played on while they talked” spoken condition, *Speech “Birthday” * “Happy Birthday to you” spoken condition,* Song “Birthday”*  “Happy Birthday to you” sung condition

Table 3 displays the confusion matrices for each emotion by condition, rounded to the nearest whole percentage.

**Table 3:** Confusion matrices for each emotion by condition, rounded to the nearest whole percentage

	Response																		
Clip Emotion	Angry	Disgusted		Embarrassed		Fearful		Happy		Hopeful		Jealous		Neutral	Proud	Sad	Sarcastic	Stressed	Surprised
Speech “Ah”																			
Angry	*45*	14		0		0		2		0		2		5	0	0	3	14	**17**
Disgusted	**8**	*61*		3		5		0		0		5		3	0	2	2	**8**	6
Embarrassed	2	**15**		*15*		14		2		0		0		11	3	8	6	14	12
Fearful	0	3		8		*40*		0		2		0		2	0	2	2	10	**32**
Happy	2	2		3		0		*33*		6		3		5	11	2	8	2	**26**
Hopeful	0	0		6		0		11		*14*		2		14	2	0	5	2	**45**
Jealous	8	17		0		2		0		2		*3*		9	0	**19**	**19**	5	17
Neutral	5	2		5		5		0		0		6		*48*	0	**12**	6	3	9
Proud	2	2		6		0		**26**		12		5		15	*8*	2	8	2	15
Sad	0	11		3		3		2		0		2		9	3	*45*	2	**14**	8
Sarcastic	2	11		5		3		0		2		5		11	0	3	*35*	3	**22**
Stressed	**15**	6		5		14		2		2		2		11	2	5	3	*25*	11
Surprised	0	2		0		2		**3**		**3**		0		**3**	2	0	2	2	*83*
Speech “Talked”																			
Angry	*55*	11		0		0		2		0		2		10	0	2	0	**18**	2
Disgusted	8	*35*		3		6		2		3		**13**		6	2	8	3	6	5
Embarrassed	3	8		*22*		8		8		5		5		9	0	**14**	2	9	8
Fearful	2	3		6		*40*		2		3		2		3	2	**16**	0	8	14
Happy	0	5		2		0		*35*		**24**		0		12	12	0	3	2	6
Hopeful	2	0		3		2		**20**		*21*		5		9	11	2	5	8	15
Jealous	14	**25**		3		2		2		3		*15*		11	0	5	8	8	6
Neutral	5	3		2		2		0		3		3		*63*	5	**13**	2	0	0
Proud	0	5		3		0		17		15		2		**27**	*23*	0	6	0	3
Sad	2	2		0		3		0		3		7		**15**	0	*63*	0	5	0
Sarcastic	8	15		5		5		0		3		11		**18**	3	3	*23*	0	6
Stressed	**13**	6		3		11		0		0		5		11	0	6	3	*29*	**13**
Surprised	2	3		**8**		6		3		6		0		**8**	6	2	0	5	*50*
Speech "Birthday"																			
Angry	*46*	11		0		2		0		0		11		10	2	2	**17**	0	0
Disgusted	8	*48*		3		2		0		0		5		0	0	8	**22**	5	2
Embarrassed	5	5		*15*		**18**		9		0		3		11	3	15	3	9	3
Fearful	0	8		6		*25*		5		5		5		**16**	0	8	5	12	6
Happy	2	0		0		0		*54*		8		0		10	**16**	0	10	0	0
Hopeful	0	0		0		8		17		*21*		2		**23**	8	3	6	3	11
Jealous	3	20		3		0		0		0		*24*		14	5	8	**24**	0	0
Neutral	8	0		0		2		5		2		**11**		*60*	0	9	5	0	0
Proud	0	2		2		0		25		6		6		11	*15*	2	**29**	2	2
Sad	2	8		0		10		2		0		5		**14**	0	*54*	2	5	0
Sarcastic	5	8		0		2		5		0		3		**12**	2	5	*58*	0	3
Stressed	12	6		0		12		3		2		5		11	0	11	**16**	*16*	6
Surprised	3	2		0		5		**18**		5		3		6	11	2	2	3	*42*
Song "Birthday"																			
Angry	*42*	**14**		4		4		0		0		7		9	0	2	7	9	4
Disgusted	7	*46*		2		**9**		0		0		6		**9**	0	7	2	4	7
Embarrassed	5	2		*11*		**21**		11		5		5		16	2	12	5	2	2
Fearful	2	2		13		*35*		2		0		4		**19**	0	15	0	6	2
Happy	0	0		0		0		*70*		6		0		4	**11**	0	6	2	2
Hopeful	0	0		6		9		**41**		*17*		6		9	7	0	0	2	4
Jealous	7	10		9		2		0		2		*7*		19	0	5	**31**	7	2
Neutral	2	0		2		7		7		2		**14**		*53*	2	9	4	0	0
Proud	2	0		4		0		**45**		9		4		18	*9*	2	7	0	0
Sad	0	2		0		9		0		0		0		**23**	0	*58*	4	5	0
Sarcastic	2	3		3		0		7		3		12		**16**	2	5	*40*	3	3
Stressed	15	2		7		15		4		6		0		**26**	2	6	7	*9*	2
Surprised	0	4		4		9		16		4		4		**21**	5	0	11	7	*16*

*Speech “Ah”* “Ah” spoken condition,* Speech “Talked”*  “The music played on while they talked” spoken condition,* Speech “Birthday”*  “Happy Birthday to you” spoken condition,* Song “Birthday”* “Happy Birthday to you” sung condition

Correct responses are italicised. The highest error percentage for each emotion by utterance is in boldface.

**Fig. 2:**
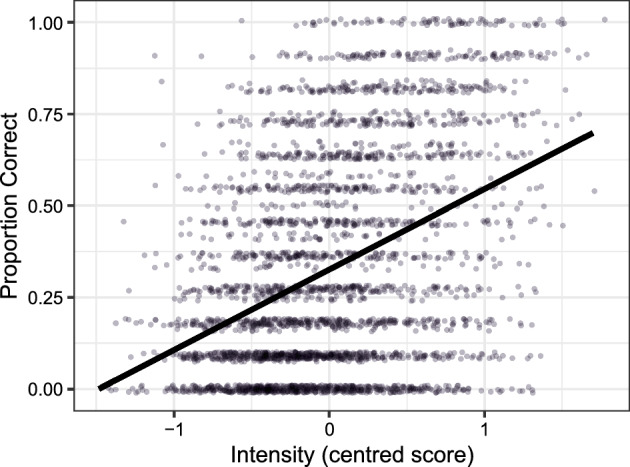
Scatter plots depicting the positive relationship between proportion correct and Intensity

We then analysed how certain characteristics of the stimuli may influence recognition accuracy of the emotion database clips using a binomial generalised linear mixed effects model (Marginal R^2^ = 0.268, Conditional R^2^ = 0.529). We found a significant main effect of Intensity (χ^2^(1) = 410.88, *p* < 0.001) but not Genuineness (χ^2^(1) = 0.47, *p* = 0.491). Based on the model parameter estimates, there was a positive relationship between recognition accuracy and Intensity (B = 1.13, SE = 0.06, *z* = 20.27, *p* < 0.001), such that the more intense the clips were rated to be, the higher the recognition accuracy of those clips (see Fig. 2).

**Fig. 3:**
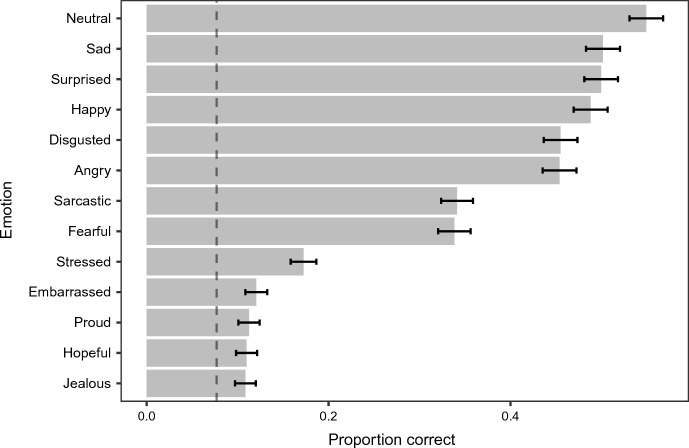
Proportion correct by emotion collapsing across conditions. Error bars represent 95% confidence intervals. Dotted line represents the theoretical chance performance (i.e., 1/13 or 0.077)

The model also revealed a significant main effect of Emotion (χ^2^(12) = 729.42, *p* < 0.001) but not Condition (χ^2^(3) = 6.33, *p* = 0.096). As can be seen from Fig. 3, the basic emotions, the Neutral emotion, and the Sarcastic emotion, were recognised more accurately than the other complex emotions. Specifically, pairwise comparisons (see Supplementary Section S4 for all the comparisons) revealed that the Neutral clips were recognised more accurately than all the other emotions other than Sad. Sad, in turn, was recognised better than all the other emotions other than Surprised and Happy. Recognition of Surprised, Happy, Disgusted, and Angry did not differ from each other. Surprised and Happy were recognised better than Sarcastic and Fearful, which in turn were recognised better than Stressed, Embarrassed, Proud, Hopeful and Jealous. Recognition of Stressed did not differ from Embarrassed, but the former was recognised better than Proud, Hopeful and Jealous.

**Fig. 4:**
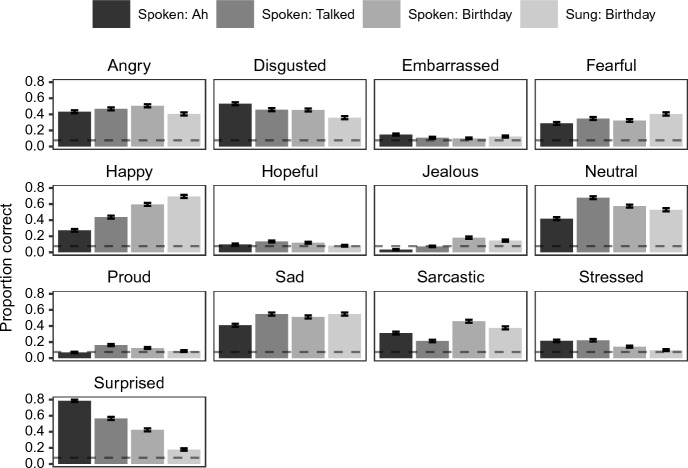
Proportion correct by emotion and condition. Error bars represent 95% confidence intervals. Dotted line represents the theoretical chance performance (i.e., 1/13 or 0.077)

The interaction between Emotion × Condition was significant (χ^2^(36) = 345.61, *p* < 0.001). To follow up the interaction, we conducted pairwise comparisons of each condition per emotion (see Fig. 4; see Supplementary Section S5 for all the comparisons). We found that, on the one hand, emotion recognition in the spoken “Ah” and/or “Talked” conditions to be better than the spoken and/or sung “Birthday” conditions for some of the emotions: (i) Disgusted was better in the spoken “Ah” condition compared to the spoken “Birthday” (*z* = 2.82, *p* = 0.025) and the sung “Birthday” (*z* = 3.68, *p* = 0.001) conditions; (ii) Embarrassed was better in spoken “Ah” than spoken “Birthday” (z = 2.76, *p* = 0.030); (iii) Neutral in the spoken “Talked” condition was recognised better than spoken “Ah” (*z* = 3.60, *p* = 0.002) and sung “Birthday” (*z* = 2.72, *p* = 0.033) conditions; (iv) Proud was recognised better in the spoken “Talked” condition than the sung “Birthday” condition (*z* = 2.74, *p* = 0.031); (v) Stressed was recognised better in the “Ah” condition than the spoken and sung “Birthday” (spoken: *z* = 2.89, *p* = 0.020; sung: *z* = 3.73, *p* = 0.001) conditions and in the “Talked” condition than the sung “Birthday” condition (*z* = 3.05, *p* = 0.012); and (vi) Surprised was recognised better in the “Ah” condition than the “Talked” condition (“Ah” vs. “Talked”: *z* = 5.38, *p* < 0.001), which in turn, outperformed the spoken “Birthday” condition (“Talked” vs. spoken “Birthday”: *z* = 2.82, *p* = 0.025) and the worst in the sung “Birthday” condition (spoken “Birthday” vs. sung “Birthday”: *z* = 4.55, *p* < 0.001). On the other hand, performance in the spoken and/or sung “Birthday” conditions were better than the spoken “Ah” and/or “Talked” conditions for three emotions: (i) Happy was recognised better in the sung and spoken “Birthday” conditions compared to the spoken “Talked” condition (sung “Birthday” vs. “Talked”: *z* = 5.02, *p* < 0.001; spoken vs. “Talked”: *z* = 3.01, *p* = 0.014), which in turn was recognised better than the spoken “Ah” condition (*z* = 3.23, *p* = 0.007); (ii) Jealous was recognised better in the spoken and sung “Birthday” conditions than the “Ah” condition (spoken “Birthday” vs. “Ah”: *z* = 4.54, *p* < 0.001; sung “Birthday” vs. “Ah”: *z* = 3.64, *p* = 0.002), and in the spoken “Birthday” than the spoken “Talked” condition (*z* = 3.46, *p* = 0.003); and (iii) Sarcastic was recognised worse in the spoken “Talked” condition than the other three conditions (“Ah” vs. “Talked”: *z* = 3.04, *p* = 0.013; spoken “Birthday” vs. “Talked”: *z* = 4.44, *p* < 0.001; sung “Birthday” vs. “Talked”: *z* = 3.39, *p* = 0.004).

**Fig. 5:**
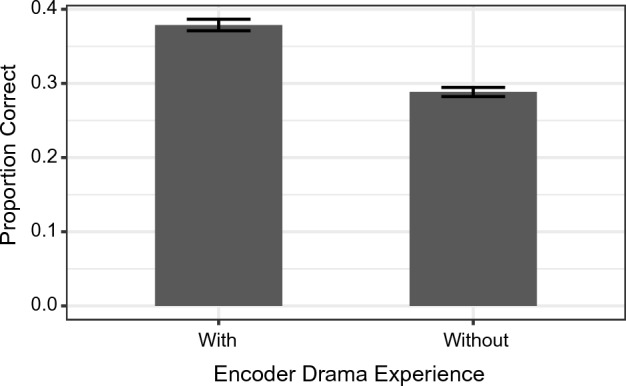
Proportion correct by Encoder Drama Experience. Error bars represent 95% confidence intervals

We conducted another mixed effects model as an exploratory analysis to examine whether individual differences in musical and drama experience among the encoders and decoders would affect recognition accuracy as a function of Condition (Marginal R^2^ = 0.013, Conditional R^2^ = 0.490). There was a significant effect of Condition (χ^2^(3) = 10.44, *p* = 0.015), such that the spoken “Birthday” condition was recognised generally better than the spoken “Ah” condition (*z* = 2.90, *p* = 0.019). No other pairwise comparisons were statistically significant. Importantly, there was a significant effect of Encoder Drama Experience (χ^2^(1) = 58.12, *p* < 0.001), such that clips produced by those with drama experience were recognised more accurately than by those without drama experience (see Fig. 5). No other effects or interactions were significant in the model.

## Discussion

We developed a new audio-visual (AV) emotion database called the Reading Everyday Emotion Database (REED) to complement those available in the literature. Specifically, unlike most previous databases that used professional actors to portray a small set of emotions in a studio-like environment, the REED consists of recordings of a wide range of emotions portrayed by everyday adult encoders (i.e., individuals with and without drama experience) using everyday recording devices (i.e., the encoders’ webcam, mobile phone, etc.). In the age where teleconferencing is prevalent, the REED thus provides a set of AV recordings of emotions that reflect such situations where variability in the recording environment is natural.

The database consists of 3230 clips across 13 different emotions in four conditions (3 spoken utterances of various lengths and 1 sung utterance). We found that, unsurprisingly, intensity ratings had a positive relationship with recognition accuracy, similar to that found in previous databases (O’Reilly et al., 2016). From the validation study, it appears that the emotions were not equally well recognised, with the basic emotions, the Neutral emotion, and the Sarcastic emotion recognised more accurately than the other complex emotions. Moreover, the results suggest that some emotions were better recognised in certain conditions. There was no difference in accuracy between the four conditions, suggesting that neither utterance length nor domain influenced recognition accuracy. Concerning utterance length, previous studies have demonstrated emotion-specific effects on recognition over the time-course of the utterance. For example, over the course of an auditory stimulus, fear tends to be recognised quickly while happiness and disgust tend to be recognised more slowly (Pell & Kotz, 2011; Rigoulot et al., 2013). Based on this, one may expect that the longer the utterance length, the more time there is for the emotional information to unfold, and therefore the better the recognition accuracy. However, above-chance emotion recognition accuracy can be achieved for auditory stimuli after just 250 ms, suggesting that emotion recognition is highly efficient (Nordström & Laukka, 2019). Thus, it may be the case that after a certain durational threshold, the utterance length will not have any effect on recognition. Alternatively, utterance length may not have any facilitative effect when information from the visual domain is also present.

Recognition accuracy was also similar between spoken and sung domains as found in our validation study. This is somewhat surprising, because even though speech and music share similar acoustic cues to express emotions (Juslin & Laukka, 2003), the encoders may be somewhat constrained by the melodic and timing properties of music when expressing sung emotions. We suspect that the lack of a difference between the two domains in this study is due to the presence of visual cues in the stimuli. Indeed, as reported previously, whereas decoders showed poorer emotion recognition accuracy in sung stimuli compared to spoken stimuli in auditory-only condition, recognition performance did not differ between the two domains in the AV condition (Livingstone et al., 2015).

When compared to some of the previous AV databases, such as the RAVDESS (Livingstone & Russo, 2018), the overall proportion correct from the REED was relatively lower. As seen in Fig. 3, depending on the specific emotion, proportion correct of the clips in the REED ranged between 0.11 and 0.55 whereas the overall proportion correct in the RAVDESS database was 0.77 (collapsing across emotions for both audio-visual speech and song at ‘normal intensity’). This difference is presumably due to several methodological factors in the RAVDESS database including the use of studio-like environment, professional actors (who, as found in the current study, produced expressions that were more easily recognised than individuals without drama experience), and only including some, but not all, of the basic emotions, which are presumably easier to recognise given their universality and their expressions are less context-dependent (Ekman, 1999; Griffiths, 1997). Indeed, with the inclusion of complex emotions (but still expressed by professional actors) in previous AV databases such as the GEMEP-CS (Bänziger et al., 2012) and the EU-Emotion (O’Reilly et al., 2016) databases, relatively lower proportion correct was found: the overall proportion correct for the GEMEP-CS database and the EU-Emotion database was 0.59 and 0.69, respectively.

The REED, while addressing several gaps in the literature, does have several limitations, with one of them being that the emotions were posed rather than spontaneously elicited by the encoders. This elicitation method was chosen to allow for a more precise control over the expression produced, that is, to establish a ‘ground truth’ of each expression by the encoder, as opposed to the possibility of encoders producing an expression that is not intended or a blend of emotions. This limitation might be exacerbated by the fact that the encoders were everyday individuals, most of whom did not have any drama or acting experience. Moreover, to increase natural variation in their expressions, we did not coach the encoders on how to produce the emotions, and so when asked to pose expressions, some of their expressions may be perceived as artificial or unnatural. We tried to circumvent this issue by measuring genuineness ratings, and as can be seen in Table 2, the mean genuineness for each emotion falls around the average range on a 5-point scale.

We tried to maximise the potential use of the REED by including various utterance lengths and having two utterances in different domains that are otherwise comparable (i.e., spoken vs. sung “Happy birthday to you”), but the utterances themselves may be confounded by its semantic content (and indeed, the Emotion × Condition interaction in our model somewhat confirms this). For example, given that “Happy birthday” is typically said and/or sung in a positive valence, it is unclear what effect this may have on the encoders’ emotion production of the other emotions, particularly those with negative valence. To explore this possibility, we conducted an acoustic analysis (reported in Supplementary Section S6) on the two spoken sentences (i.e., “Happy Birthday to you” and “The music played on while they talked”). We found that there is an influence of utterance on the mean pitch, mean intensity, and duration of the emotional expressions, but the findings are contrary to the predictions of any ‘carry-over’ positive-valenced effect of the semantic content or context of “Happy Birthday to you”. That is, assuming that there is a carry-over effect, one might expect that the semantically-positive “Happy Birthday to you” utterance would have higher mean pitch, higher mean intensity, and shorter duration relative to the semantically-neutral “The music played on while they talked” utterance on all the emotions (given that the former utterance is typically expressed in a Happy or Surprised emotion). This was not the case in the acoustic analysis: in fact, the “birthday” utterance had lower mean pitch and intensity generally, and shorter duration (despite normalisation to account for syllabic differences) in only two of the emotions than the “talked” utterance. Moreover, the semantic content of the utterances does not fully explain the Emotion × Condition interaction found in the recognition task, as there were recognition differences between conditions for emotions that are not immediately clear (e.g., recognition performance was better in the spoken and sung “Birthday” conditions than the spoken “Ah” condition for Jealous and the spoken “Talked” condition for Sarcastic). Thus, it seems that while the different utterances do differ acoustically and in their subsequent recognition, this may not be due solely to the semantic content or context of the utterances per se. Further research is needed to clarify whether the semantic content does indeed influence the emotional expression and, if so, in what ways.

In conclusion, in this paper, we described a new AV database called the Reading Everyday Emotion Database (REED) that consists of a wide range of emotions spoken and sung by everyday individuals using everyday recording devices, complementing previous databases that typically use professional actors in a studio-like environment. Clips in the REED have been validated by a separate group of participants. Despite some of the limitations highlighted, we believe that the REED will be useful for those that require audio-visual clips to have natural variations in the encoders’ expressions and in the recording environment. The complete REED database is available to authorised users subject to a Data Access Agreement, which can be accessed at the following link: https://doi.org/10.17864/1947.000407.

## Supplementary Information

Below is the link to the electronic supplementary material.

Supplementary file1 (DOCX 594 KB)

## Data Availability

The datasets generated in the validation study are available in the University of Reading Data Archive, https://doi.org/10.17864/1947.000407. The complete REED database is available to authorised users subject to a Data Access Agreement, which can be accessed at the following link: https://doi.org/10.17864/1947.000407.

## Footnotes

1 The ‘Reading’ in REED is pronounced as ‘Redding’, following the town in Berkshire, England, where the university is located.

2 Due to the coronavirus (COVID-19) pandemic, we were not able to systematically manipulate the device and recording conditions for each encoder in the lab but instead, relied on each encoder’s own recording environment for variation.

3 The encoders were asked to report whether they have had any formal musical/vocal training, and if so, on what instrument, for how long, and whether they were still practising. Full details about encoders’ musical background can be found in Supplementary Section S1.

4 For the purpose of this study and for convenience, we classify “Ah” as speech (or specifically, not song) though we recognise that some may consider it to be a nonverbal utterance (Belin et al., 2008).

5 The scenarios were validated by four participants who were not involved in any aspects of the project by choosing the most appropriate emotion for each scenario.

6 Due to our data sharing policy, we are unable to share screenshots of all the encoders unless users have signed the Data Access Agreement form. See Supplementary Section S3 for a figure displaying a portion of the screenshots of all the encoders in REED to get a sense of variability in the recording quality of the clips.
