# Supplementary material for: The Reading Everyday Emotion Database (REED): a set of audio-visual recordings of emotions in music and language
Source: Lang Resour Eval. 2023 Nov 20;59(1):27–49. doi: 10.1007/s10579-023-09698-5 (PMC11913894; doi:10.1007/s10579-023-09698-5)
Supplement: Supplementary file 1 — Supplementary file1 (DOCX 594 KB) [file 10579_2023_9698_MOESM1_ESM.docx]

**The Reading Everyday Emotion Database (REED):**

**A set of audio-visual recordings of emotions in music and language**

Jia Hoong Ong^1,2^, Florence Yik Nam Leung^1,3^, Fang Liu^1,a^

^1^ School of Psychology and Clinical Language Sciences, University of Reading, Reading, United Kingdom

^2^ Department of Psychology, School of Social Sciences, Nottingham Trent University, Nottingham, United Kingdom

^3^ Department of Psychology, University of Bath, Bath, United Kingdom

^a^ Corresponding author. Email: [f.liu@reading.ac.uk](mailto:f.liu@reading.ac.uk); Telephone: +44 (0)118 378 8122; Mailing address: Harry Pitt Building, School of Psychology & Clinical Language Sciences, University of Reading, Earley Gate, Reading RG6 6AL

**SUPPLEMENTARY SECTIONS**

[Section S1: Encoder demographic details](#_Section_S1:_Encoder)

[Section S2: Scenarios](#_Section_S2:_Scenarios)

[Section S3: Screenshots of recording clips](#_Section_S3:_Screenshots_1)

[Section S4: Pairwise comparisons of Emotion](#_Section_S4:_Pairwise)

[Section S5: Pairwise comparisons of Condition by Emotion](#_Section_S5:_Pairwise)

[Section S6: Acoustic analysis](#_Section_S6:_Acoustic)

# Section S1: Encoder demographic details

**Table S1.1**

*Details of each encoder (Code) and their demographic details including Age; Gender; their musical training experience (MT); the cumulative years of musical training across multiple instruments (MT Years); whether they were still practising music (MT Still); their drama experience (Drama Exp); and their recording devices (Device).*

| **Code** | **Age** | **Gender** | **MT** | **MT Years** | **MT Still** | **Drama Exp** | **Device** |
| --- | --- | --- | --- | --- | --- | --- | --- |
| FW01 | 21 | F | Self-taught, piano, 4 years; self-taught, guitar, 4 years | 8 | No | NA | Macbook Air (2013) |
| FW02 | 23 | F | Private lessons, piano, 8 years; Private lessons, violin, 5 years | 13 | Yes | NA | Lenovo V110 |
| FW03 | 25 | F | Private lessons, Piano, 5 years | 5 | No | NA | HP Pavillion 14 |
| FW04 | 25 | F | Private lessons, Piano, 6 years; Group lessons, Voice, 12 years | 18 | Yes | Professional actress in film and on stage. Member of the National Youth Theatre of Great Britain. | iPad Pro (2018) |
| FW05 | 26 | F | Private lessons, Piano, 5 years | 5 | No | NA | Macbook Pro (2012) |
| FW06 | 22 | F | NA | NA | NA | NA | Macbook Pro (2019) |
| FW07 | 75 | F | Private lessons, French horn, 6 years | 6 | Yes | NA | Toshiba Satellite C855 |
| FW08 | 45 | F | Group lessons, keyboard, 2 years; Private lessons, violin, 5 years | 7 | No | NA | HP Pavilion G6 |
| FW10 | 81 | F | NA | NA | NA | Acting/directing at a local theatre. Exams - LAMDA Bronze and Silver | Mac laptop (2017/2018) |
| FW11 | 39 | F | NA | NA | NA | Drama with dance degree 2003, PGCE Drama Teacher 2003. Taught drama ever since and been part of 2 amateur dramatics theatres within that time. | Microsoft Surface Pro |
| FW12 | 31 | F | Private lessons, Piano, 22 years; Private lessons, Electric Organ, 12 years; Group lessons, Singing, 18 years | 52 | Yes | Performed in musical theatre | iPhone 8 |
| FW13 | 38 | F | NA | NA | NA | NA | Dell 7400 |
| MW01 | 22 | M | Self-taught, Guitar, 9 years | 9 | Yes | GCSE Drama | Lenovo Thinkpad T440s |
| MW02 | 33 | M | Private lessons, Piano, 7 years | 7 | Yes | NA | HP 3168NGW |
| MW03 | 75 | M | NA | NA | NA | Appeared in several amateur plays in their youth | Toshiba Satellite C855 |
| MW04 | 60 | M | NA | NA | NA | Did a little professional work when younger now frequently do amateur productions | iPad Air (2020) |
| MW05 | 24 | M | Private lessons, Clarinet, 9 years | 9 | No | Performed in multiple amateur/university society productions and festivals | MacBook Pro (2017) |
| MW06 | 33 | M | Private lessons, Piano, 26 years | 26 | Yes | Practised from school until one year after university | MacBook Air (mid 2013) |
| MW09 | 20 | M | NA | NA | NA | NA | iPhone XS Max |
| MW10 | 19 | M | NA | NA | NA | NA | Samsung Galaxy S20 |
| MW11 | 50 | M | NA | NA | NA | NA | Dell Ultrabook Latitude E7270 |
| MW12 | 53 | M | NA | NA | NA | NA | MacBook Air 13” (2017) |

# Section S2: Scenarios

**Table S2.1**

*List of emotions, definitions, and the scenarios given to the encoders to be used for eliciting expressions.*

| **Emotion** | **Definition** | **Scenario** |
| --- | --- | --- |
| Neutral | Dispassionate, detached | You just finished meditating. |
| Stressed | Pressured, worried | You have been told that you have to completely rewrite your essay but it is due in less than an hour. |
| Proud | Fulfilling, satisfied | You worked tirelessly to complete a project and it is done and it is well received. |
| Disgusted | Revulsion, sicken | You’re just about to eat a bite of your food but then you noticed maggots on it. |
| Happy | Joyous, blissful | You’re on a holiday to a place that you have always wanted to visit. |
| Hopeful | Optimistic, positive | You think your favourite football team has a chance of winning the championship title this year based on their past few games. |
| Jealous | Envious, resentful | Your younger brother got the latest gaming console that you wanted for Christmas whereas you got a pair of socks. |
| Fearful | Terrified, worried | You’re alone at home at night and you hear some noise coming from the kitchen. |
| Sad | Heavy-hearted, sorrowful | Your pet of 10 years has to be put down because of illness. |
| Surprised | Unexpected, astonished | The movie you’re watching has a plot twist that you did not see coming |
| Sarcastic | Cynical, mocking | When someone asks how your terrible day is going, so you respond "Today cannot get any better". |
| Embarrassed | Ashamed, humiliated | You just did a presentation in front of your boss and colleagues, and then you realised you had on a shirt with a massive food stain. |
| Angry | Furious, cross | You’re losing your temper because you were crossing a zebra crossing when a car doesn’t give way and narrowly misses you. |

#

# Section S3: Screenshots of recording clips

**Figure S3.1**

*A portion of screenshots of all the encoders in the REED. The first two columns represent screenshots of female encoders whereas the last two columns represent screenshots of male encoders.*

# Section S4: Pairwise comparisons of Emotion

**Table S4.1**

*Pairwise comparisons of Emotion.*

| **Contrast 1** | **Contrast 2** | ***z*** | ***p*** |
| --- | --- | --- | --- |
| Angry | Disgusted | 0.17 | 1.000 |
| Angry | Embarrassed | 10.93 | < .001 |
| Angry | Fearful | 2.95 | .140 |
| Angry | Happy | -1.31 | .985 |
| Angry | Hopeful | 12.23 | < .001 |
| Angry | Jealous | 13.08 | < .001 |
| Angry | Neutral | -7.05 | < .001 |
| Angry | Proud | 13.20 | < .001 |
| Angry | Sad | -4.23 | .002 |
| Angry | Sarcastic | 2.95 | .140 |
| Angry | Stressed | 9.38 | < .001 |
| Angry | Surprised | -1.06 | .998 |
| Disgusted | Embarrassed | 11.05 | < .001 |
| Disgusted | Fearful | 2.78 | .213 |
| Disgusted | Happy | -1.37 | .979 |
| Disgusted | Hopeful | 12.74 | < .001 |
| Disgusted | Jealous | 13.18 | < .001 |
| Disgusted | Neutral | -7.08 | < .001 |
| Disgusted | Proud | 13.63 | < .001 |
| Disgusted | Sad | -4.22 | .002 |
| Disgusted | Sarcastic | 2.69 | .260 |
| Disgusted | Stressed | 9.53 | < .001 |
| Disgusted | Surprised | -1.20 | .993 |
| Embarrassed | Fearful | -8.91 | < .001 |
| Embarrassed | Happy | -11.29 | < .001 |
| Embarrassed | Hopeful | 1.28 | .988 |
| Embarrassed | Jealous | 2.43 | .421 |
| Embarrassed | Neutral | -16.56 | < .001 |
| Embarrassed | Proud | 2.54 | .350 |
| Embarrassed | Sad | -14.28 | < .001 |
| Embarrassed | Sarcastic | -9.00 | < .001 |
| Embarrassed | Stressed | -2.26 | .547 |
| Embarrassed | Surprised | -12.19 | < .001 |
| Fearful | Happy | -3.89 | .007 |
| Fearful | Hopeful | 10.45 | < .001 |
| Fearful | Jealous | 11.48 | < .001 |
| Fearful | Neutral | -9.62 | < .001 |
| Fearful | Proud | 11.24 | < .001 |
| Fearful | Sad | -6.79 | < .001 |
| Fearful | Sarcastic | -0.07 | 1.000 |
| Fearful | Stressed | 7.03 | < .001 |
| Fearful | Surprised | -3.86 | .007 |
| Happy | Hopeful | 12.25 | < .001 |
| Happy | Jealous | 12.96 | < .001 |
| Happy | Neutral | -5.55 | < .001 |
| Happy | Proud | 12.37 | < .001 |
| Happy | Sad | -2.53 | .356 |
| Happy | Sarcastic | 3.87 | .007 |
| Happy | Stressed | 9.80 | < .001 |
| Happy | Surprised | 0.30 | 1.000 |
| Hopeful | Jealous | 1.29 | .987 |
| Hopeful | Neutral | -17.46 | < .001 |
| Hopeful | Proud | 1.12 | .996 |
| Hopeful | Sad | -15.89 | < .001 |
| Hopeful | Sarcastic | -10.72 | < .001 |
| Hopeful | Stressed | -3.60 | .019 |
| Hopeful | Surprised | -13.53 | < .001 |
| Jealous | Neutral | -17.80 | < .001 |
| Jealous | Proud | -0.05 | 1.000 |
| Jealous | Sad | -16.76 | < .001 |
| Jealous | Sarcastic | -11.56 | < .001 |
| Jealous | Stressed | -4.76 | < .001 |
| Jealous | Surprised | -14.23 | < .001 |
| Neutral | Proud | 17.68 | < .001 |
| Neutral | Sad | 3.07 | .101 |
| Neutral | Sarcastic | 9.80 | < .001 |
| Neutral | Stressed | 16.16 | < .001 |
| Neutral | Surprised | 5.93 | < .001 |
| Proud | Sad | -16.10 | < .001 |
| Proud | Sarcastic | -11.20 | < .001 |
| Proud | Stressed | -4.59 | < .001 |
| Proud | Surprised | -13.97 | < .001 |
| Sad | Sarcastic | 6.86 | < .001 |
| Sad | Stressed | 13.17 | < .001 |
| Sad | Surprised | 2.97 | .132 |
| Sarcastic | Stressed | 7.51 | < .001 |
| Sarcastic | Surprised | -4.00 | .004 |
| Stressed | Surprised | -10.18 | < .001 |

# Section S5: Pairwise comparisons of Condition by Emotion

**Table S5.1**

*Pairwise comparisons of Condition for each Emotion.*

| **Emotion** | **Contrast 1** | **Contrast 2** | ***z*** | ***p*** |
| --- | --- | --- | --- | --- |
| Angry | sung "Birthday" | spoken "Ah" | -1.47 | .457 |
| Angry | sung "Birthday" | spoken "Birthday" | -1.20 | .625 |
| Angry | sung "Birthday" | spoken “Talked” | -1.24 | .600 |
| Angry | spoken "Ah" | spoken "Birthday" | 0.26 | .994 |
| Angry | spoken "Ah" | spoken “Talked” | 0.20 | .997 |
| Angry | spoken "Birthday" | spoken “Talked” | -0.05 | 1.000 |
| Disgusted | sung "Birthday" | spoken "Ah" | -3.68 | .001 |
| Disgusted | sung "Birthday" | spoken "Birthday" | -1.01 | .746 |
| Disgusted | sung "Birthday" | spoken “Talked” | -1.71 | .317 |
| Disgusted | spoken "Ah" | spoken "Birthday" | 2.82 | .025 |
| Disgusted | spoken "Ah" | spoken “Talked” | 2.07 | .165 |
| Disgusted | spoken "Birthday" | spoken “Talked” | -0.76 | .874 |
| Embarrassed | sung "Birthday" | spoken "Ah" | -1.40 | .501 |
| Embarrassed | sung "Birthday" | spoken "Birthday" | 1.25 | .595 |
| Embarrassed | sung "Birthday" | spoken “Talked” | 0.35 | .985 |
| Embarrassed | spoken "Ah" | spoken "Birthday" | 2.76 | .030 |
| Embarrassed | spoken "Ah" | spoken “Talked” | 1.81 | .269 |
| Embarrassed | spoken "Birthday" | spoken “Talked” | -0.93 | .789 |
| Fearful | sung "Birthday" | spoken "Ah" | 2.11 | .152 |
| Fearful | sung "Birthday" | spoken "Birthday" | 1.69 | .331 |
| Fearful | sung "Birthday" | spoken “Talked” | 1.56 | .400 |
| Fearful | spoken "Ah" | spoken "Birthday" | -0.48 | .964 |
| Fearful | spoken "Ah" | spoken “Talked” | -0.60 | .934 |
| Fearful | spoken "Birthday" | spoken “Talked” | -0.12 | .999 |
| Happy | sung "Birthday" | spoken "Ah" | 7.95 | < .001 |
| Happy | sung "Birthday" | spoken "Birthday" | 2.13 | .143 |
| Happy | sung "Birthday" | spoken “Talked” | 5.02 | < .001 |
| Happy | spoken "Ah" | spoken "Birthday" | -6.10 | < .001 |
| Happy | spoken "Ah" | spoken “Talked” | -3.23 | .007 |
| Happy | spoken "Birthday" | spoken “Talked” | 3.01 | .014 |
| Hopeful | sung "Birthday" | spoken "Ah" | -0.95 | .777 |
| Hopeful | sung "Birthday" | spoken "Birthday" | -1.65 | .350 |
| Hopeful | sung "Birthday" | spoken “Talked” | -2.06 | .166 |
| Hopeful | spoken "Ah" | spoken "Birthday" | -0.71 | .892 |
| Hopeful | spoken "Ah" | spoken “Talked” | -1.13 | .671 |
| Hopeful | spoken "Birthday" | spoken “Talked” | -0.42 | .975 |
| Jealous | sung "Birthday" | spoken "Ah" | 3.64 | .002 |
| Jealous | sung "Birthday" | spoken "Birthday" | -0.94 | .783 |
| Jealous | sung "Birthday" | spoken “Talked” | 2.42 | .073 |
| Jealous | spoken "Ah" | spoken "Birthday" | -4.54 | < .001 |
| Jealous | spoken "Ah" | spoken “Talked” | -1.45 | .471 |
| Jealous | spoken "Birthday" | spoken “Talked” | 3.46 | .003 |
| Neutral | sung "Birthday" | spoken "Ah" | 0.85 | .832 |
| Neutral | sung "Birthday" | spoken "Birthday" | -0.77 | .868 |
| Neutral | sung "Birthday" | spoken “Talked” | -2.72 | .033 |
| Neutral | spoken "Ah" | spoken "Birthday" | -1.66 | .345 |
| Neutral | spoken "Ah" | spoken “Talked” | -3.60 | .002 |
| Neutral | spoken "Birthday" | spoken “Talked” | -2.03 | .177 |
| Proud | sung "Birthday" | spoken "Ah" | -0.30 | .991 |
| Proud | sung "Birthday" | spoken "Birthday" | -1.48 | .450 |
| Proud | sung "Birthday" | spoken “Talked” | -2.74 | .031 |
| Proud | spoken "Ah" | spoken "Birthday" | -1.18 | .640 |
| Proud | spoken "Ah" | spoken “Talked” | -2.47 | .066 |
| Proud | spoken "Birthday" | spoken “Talked” | -1.37 | .519 |
| Sad | sung "Birthday" | spoken "Ah" | 1.08 | .701 |
| Sad | sung "Birthday" | spoken "Birthday" | 0.42 | .975 |
| Sad | sung "Birthday" | spoken “Talked” | 0.09 | 1.000 |
| Sad | spoken "Ah" | spoken "Birthday" | -0.67 | .907 |
| Sad | spoken "Ah" | spoken “Talked” | -1.01 | .741 |
| Sad | spoken "Birthday" | spoken “Talked” | -0.35 | .986 |
| Sarcastic | sung "Birthday" | spoken "Ah" | 0.40 | .979 |
| Sarcastic | sung "Birthday" | spoken "Birthday" | -0.96 | .771 |
| Sarcastic | sung "Birthday" | spoken “Talked” | 3.39 | .004 |
| Sarcastic | spoken "Ah" | spoken "Birthday" | -1.38 | .510 |
| Sarcastic | spoken "Ah" | spoken “Talked” | 3.04 | .013 |
| Sarcastic | spoken "Birthday" | spoken “Talked” | 4.44 | < .001 |
| Stressed | sung "Birthday" | spoken "Ah" | -3.73 | .001 |
| Stressed | sung "Birthday" | spoken "Birthday" | -1.06 | .715 |
| Stressed | sung "Birthday" | spoken “Talked” | -3.05 | .012 |
| Stressed | spoken "Ah" | spoken "Birthday" | 2.89 | .020 |
| Stressed | spoken "Ah" | spoken “Talked” | 0.76 | .871 |
| Stressed | spoken "Birthday" | spoken “Talked” | -2.15 | .139 |
| Surprised | sung "Birthday" | spoken "Ah" | -11.89 | < .001 |
| Surprised | sung "Birthday" | spoken "Birthday" | -4.55 | < .001 |
| Surprised | sung "Birthday" | spoken “Talked” | -7.06 | < .001 |
| Surprised | spoken "Ah" | spoken "Birthday" | 8.20 | < .001 |
| Surprised | spoken "Ah" | spoken “Talked” | 5.38 | < .001 |
| Surprised | spoken "Birthday" | spoken “Talked” | -2.82 | .025 |

# Section S6: Acoustic analysis

One concern for using the utterance “Happy Birthday to you” is the possibility that the semantic content and the context in which the utterance is typically expressed (e.g., happy and/or surprised) may have affected the speaker’s emotional expression of that utterance. Happy and Surprised speech are typically characterised by having higher frequency (i.e., higher pitch), higher intensity (i.e., louder) and are faster (i.e., shorter in duration) (Juslin & Laukka, 2003; Livingstone et al., 2013; Sobin & Alpert, 1999). If there is an influence of the semantic content and/or the context, then we would expect that that the spoken “Happy Birthday to you” utterance will exhibit those characteristics, regardless of the emotion it is supposed to express. The goal for this acoustic analysis, thus, is to determine whether there is such an influence by directly comparing the acoustic parameters of the two spoken sentences in the REED: “Happy Birthday to you” (hereafter “birthday”) and “The music played on while they talked” (hereafter “talked”).

For each clip of the two utterances, we obtained three acoustic parameters using the Praat script ProsodyPro (Xu, 2013): mean pitch, mean intensity, and duration. Mean pitch and mean intensity were mean-centred by speaker to account for speaker-specific idiosyncrasies in their speech. For example, the mean pitches for Speaker 1 may be 0 for Emotion A (i.e., the average pitch for Speaker 1) but -10 for Emotion B (i.e., lower than Speaker 1’s average pitch by 10 Hz). Duration was mean-centred by speaker and also by utterance, to account for both speaker-specific idiosyncrasies and differences in the number of syllables between the two utterances.

Data analysis was conducted using R (R Core Team, 2021). We fitted a linear regression model using the *lm* function for each acoustic parameter, with the acoustic parameter as the dependent variable, and Utterance, Emotion and the interaction between the two as predictors. Statistical significance of the predictors was determined using the *Anova* function from the *car* package (Fox & Weisberg, 2019). Posthoc comparisons were conducted using the *emmeans* package (Lenth, 2019). Results of each of the models are reported in the subsections below.

## Mean Pitch

Unsurprisingly, the mean pitch differed by Emotion (*F*(12, 1637) = 23.89, *p* < .001) – see Figure S6.1. The mean pitch for Surprised were significantly higher than the other emotions, and the mean pitch for Fearful, Hopeful, Happy, and Angry, were significantly higher than Neutral, Embarrassed, Jealous, Sad, and Sarcastic (see Table S6.1 for all the pairwise comparisons, which is placed at the end of this section for ease of readability). There was also a main effect of Utterance (*F*(1, 1637) = 5.95, *p* = .015), such that the mean pitch for the “talked” utterance (M = 11.50, SE = 1.90) was significantly higher than that for the “birthday” utterance (M = 4.91, SE = 1.89). Thus, the semantic content of the utterance does seem to influence mean pitch, but not in the direction that is expected (i.e., if there is a carry-over positive-valenced effect of the semantic content or context of “happy birthday”, the “birthday” utterance should have higher pitch generally). There was no Emotion × Utterance interaction (*F*(12, 1637) = 1.43, *p* = .145).

**Figure S6.1**

*Mean pitch (in Hz, normalised by speaker) for each Emotion.*

## Mean Intensity

The mean intensity also differed by Emotion (*F*(12, 1637) = 19.33, *p* < .001), as depicted in Figure S6.2. Generally, the mean intensity for Angry, Surprised, Happy, Stressed, and Hopeful were not significantly different from each other, but were higher than most of the other emotions (see Table S6.2 at the end of this section for all the pairwise comparisons). We also found a main effect of Utterance (*F*(1, 1637) = 74.12, *p* < .001), with the mean intensity for the “talked” utterance (M = 0.41, SE = 0.11) being higher than that for the “birthday” utterance (M = -0.86, SE = 0.10). Again, this seems counterintuitive if there is an influence of semantic content or context for the “birthday” utterance on emotional expression (i.e., if true, the “birthday” utterance should have higher intensity generally). There was no Emotion × Utterance interaction (*F*(12, 1637) = 0.27, *p* = .994).

**Figure S6.2**

*Mean intensity (in dB, normalised by speaker) for each Emotion.*

## Duration

Though there was no main effect of Utterance on the duration of the clips (*F*(1, 1637) = 0.01, *p* = .928), there was a main effect of Emotion (*F*(12, 1637) = 17.59, *p* < .001), which was qualified by a significant Emotion × Utterance interaction (*F*(12, 1637) = 2.90, *p* < .001), as depicted in Figure S6.3. Comparisons between utterances for each emotion revealed that the duration for the “talked” utterance was significantly longer than the “birthday” utterance for Fearful (*t*(1637) = 2.36, *p* = .019) and Sad (*t*(1637) = 4.34, *p* < .001), but the duration for the “birthday” utterance was significantly longer than the “talked” utterance for Surprised (*t*(1637) = 1.97, *p* = .049). See Table S6.3 at the end of this section for all the comparisons. Thus, as with the findings for mean pitch and mean intensity, this is mostly incompatible with the expectations that if there was an influence of semantic content or context of the emotional expression of the “birthday” utterance (i.e., if true, the “birthday” utterance should be shorter, which was found to be true in only two emotions, and not true for the emotion that we expect it to be).

**Figure S6.3**

*Duration (in ms, normalised by speaker and utterance) for each Emotion by Utterance.*

**Table S6.1**

*Pairwise comparisons of Emotion for mean pitch.*

| **Contrast 1** | **Contrast 2** | ***t*** | ***p*** |
| --- | --- | --- | --- |
| Angry | Disgusted | 1.11 | .997 |
| Angry | Embarrassed | 4.59 | < .001 |
| Angry | Fearful | -0.75 | 1.000 |
| Angry | Happy | -0.09 | 1.000 |
| Angry | Hopeful | -0.34 | 1.000 |
| Angry | Jealous | 4.38 | .001 |
| Angry | Neutral | 6.37 | < .001 |
| Angry | Proud | 2.97 | .133 |
| Angry | Sad | 4.17 | .002 |
| Angry | Sarcastic | 3.87 | .007 |
| Angry | Stressed | 0.66 | 1.000 |
| Angry | Surprised | -6.99 | < .001 |
| Disgusted | Embarrassed | 3.49 | .028 |
| Disgusted | Fearful | -1.87 | .811 |
| Disgusted | Happy | -1.20 | .993 |
| Disgusted | Hopeful | -1.47 | .962 |
| Disgusted | Jealous | 3.28 | .056 |
| Disgusted | Neutral | 5.29 | < .001 |
| Disgusted | Proud | 1.86 | .816 |
| Disgusted | Sad | 3.08 | .099 |
| Disgusted | Sarcastic | 2.78 | .214 |
| Disgusted | Stressed | -0.46 | 1.000 |
| Disgusted | Surprised | -8.15 | < .001 |
| Embarrassed | Fearful | -5.36 | < .001 |
| Embarrassed | Happy | -4.69 | < .001 |
| Embarrassed | Hopeful | -5.00 | < .001 |
| Embarrassed | Jealous | -0.23 | 1.000 |
| Embarrassed | Neutral | 1.82 | .841 |
| Embarrassed | Proud | -1.65 | .915 |
| Embarrassed | Sad | -0.37 | 1.000 |
| Embarrassed | Sarcastic | -0.71 | 1.000 |
| Embarrassed | Stressed | -3.94 | .006 |
| Embarrassed | Surprised | -11.66 | < .001 |
| Fearful | Happy | 0.67 | 1.000 |
| Fearful | Hopeful | 0.42 | 1.000 |
| Fearful | Jealous | 5.16 | < .001 |
| Fearful | Neutral | 7.15 | < .001 |
| Fearful | Proud | 3.74 | .012 |
| Fearful | Sad | 4.93 | < .001 |
| Fearful | Sarcastic | 4.64 | < .001 |
| Fearful | Stressed | 1.42 | .972 |
| Fearful | Surprised | -6.26 | < .001 |
| Happy | Hopeful | -0.26 | 1.000 |
| Happy | Jealous | 4.48 | .001 |
| Happy | Neutral | 6.48 | < .001 |
| Happy | Proud | 3.07 | .103 |
| Happy | Sad | 4.27 | .002 |
| Happy | Sarcastic | 3.97 | .005 |
| Happy | Stressed | 0.75 | 1.000 |
| Happy | Surprised | -6.93 | < .001 |
| Hopeful | Jealous | 4.79 | < .001 |
| Hopeful | Neutral | 6.81 | < .001 |
| Hopeful | Proud | 3.36 | .044 |
| Hopeful | Sad | 4.57 | < .001 |
| Hopeful | Sarcastic | 4.27 | .001 |
| Hopeful | Stressed | 1.01 | .999 |
| Hopeful | Surprised | -6.74 | < .001 |
| Jealous | Neutral | 2.05 | .699 |
| Jealous | Proud | -1.42 | .971 |
| Jealous | Sad | -0.15 | 1.000 |
| Jealous | Sarcastic | -0.49 | 1.000 |
| Jealous | Stressed | -3.73 | .012 |
| Jealous | Surprised | -11.48 | < .001 |
| Neutral | Proud | -3.46 | .031 |
| Neutral | Sad | -2.16 | .618 |
| Neutral | Sarcastic | -2.52 | .361 |
| Neutral | Stressed | -5.74 | < .001 |
| Neutral | Surprised | -13.43 | < .001 |
| Proud | Sad | 1.26 | .990 |
| Proud | Sarcastic | 0.93 | .999 |
| Proud | Stressed | -2.32 | .504 |
| Proud | Surprised | -10.06 | < .001 |
| Sad | Sarcastic | -0.33 | 1.000 |
| Sad | Stressed | -3.53 | .025 |
| Sad | Surprised | -11.15 | < .001 |
| Sarcastic | Stressed | -3.23 | .066 |
| Sarcastic | Surprised | -10.92 | < .001 |
| Stressed | Surprised | -7.68 | < .001 |

**Table S6.2**

*Pairwise comparisons of Emotion for mean intensity.*

| **Contrast 1** | **Contrast 2** | ***t*** | ***p*** |
| --- | --- | --- | --- |
| Angry | Disgusted | 5.16 | < .001 |
| Angry | Embarrassed | 7.11 | < .001 |
| Angry | Fearful | 6.14 | < .001 |
| Angry | Happy | 1.04 | .998 |
| Angry | Hopeful | 2.76 | .221 |
| Angry | Jealous | 7.22 | < .001 |
| Angry | Neutral | 7.20 | < .001 |
| Angry | Proud | 4.38 | .001 |
| Angry | Sad | 9.89 | < .001 |
| Angry | Sarcastic | 4.47 | .001 |
| Angry | Stressed | 1.33 | .984 |
| Angry | Surprised | 0.48 | 1.000 |
| Disgusted | Embarrassed | 1.95 | .766 |
| Disgusted | Fearful | 0.99 | .999 |
| Disgusted | Happy | -4.13 | .003 |
| Disgusted | Hopeful | -2.45 | .408 |
| Disgusted | Jealous | 2.04 | .707 |
| Disgusted | Neutral | 2.06 | .693 |
| Disgusted | Proud | -0.81 | 1.000 |
| Disgusted | Sad | 4.81 | < .001 |
| Disgusted | Sarcastic | -0.70 | 1.000 |
| Disgusted | Stressed | -3.85 | .008 |
| Disgusted | Surprised | -4.70 | < .001 |
| Embarrassed | Fearful | -0.95 | .999 |
| Embarrassed | Happy | -6.09 | < .001 |
| Embarrassed | Hopeful | -4.42 | .001 |
| Embarrassed | Jealous | 0.08 | 1.000 |
| Embarrassed | Neutral | 0.12 | 1.000 |
| Embarrassed | Proud | -2.77 | .216 |
| Embarrassed | Sad | 2.89 | .165 |
| Embarrassed | Sarcastic | -2.65 | .284 |
| Embarrassed | Stressed | -5.80 | < .001 |
| Embarrassed | Surprised | -6.66 | < .001 |
| Fearful | Happy | -5.12 | < .001 |
| Fearful | Hopeful | -3.45 | .033 |
| Fearful | Jealous | 1.04 | .998 |
| Fearful | Neutral | 1.07 | .998 |
| Fearful | Proud | -1.81 | .845 |
| Fearful | Sad | 3.82 | .009 |
| Fearful | Sarcastic | -1.69 | .900 |
| Fearful | Stressed | -4.83 | < .001 |
| Fearful | Surprised | -5.69 | < .001 |
| Happy | Hopeful | 1.72 | .887 |
| Happy | Jealous | 6.19 | < .001 |
| Happy | Neutral | 6.18 | < .001 |
| Happy | Proud | 3.35 | .046 |
| Happy | Sad | 8.89 | < .001 |
| Happy | Sarcastic | 3.44 | .034 |
| Happy | Stressed | 0.29 | 1.000 |
| Happy | Surprised | -0.56 | 1.000 |
| Hopeful | Jealous | 4.52 | .001 |
| Hopeful | Neutral | 4.52 | .001 |
| Hopeful | Proud | 1.65 | .915 |
| Hopeful | Sad | 7.27 | < .001 |
| Hopeful | Sarcastic | 1.75 | .874 |
| Hopeful | Stressed | -1.43 | .970 |
| Hopeful | Surprised | -2.29 | .525 |
| Jealous | Neutral | 0.04 | 1.000 |
| Jealous | Proud | -2.87 | .173 |
| Jealous | Sad | 2.82 | .195 |
| Jealous | Sarcastic | -2.74 | .233 |
| Jealous | Stressed | -5.90 | < .001 |
| Jealous | Surprised | -6.77 | < .001 |
| Neutral | Proud | -2.88 | .167 |
| Neutral | Sad | 2.76 | .223 |
| Neutral | Sarcastic | -2.75 | .225 |
| Neutral | Stressed | -5.89 | < .001 |
| Neutral | Surprised | -6.75 | < .001 |
| Proud | Sad | 5.64 | < .001 |
| Proud | Sarcastic | 0.11 | 1.000 |
| Proud | Stressed | -3.06 | .106 |
| Proud | Surprised | -3.92 | .006 |
| Sad | Sarcastic | -5.50 | < .001 |
| Sad | Stressed | -8.61 | < .001 |
| Sad | Surprised | -9.46 | < .001 |
| Sarcastic | Stressed | -3.15 | .082 |
| Sarcastic | Surprised | -4.01 | .004 |
| Stressed | Surprised | -0.85 | 1.000 |

**Table S6.3**

*Pairwise comparisons on duration between utterances for each Emotion.*

| **Utterance** | **Emotion** | ***t*** | ***p*** |
| --- | --- | --- | --- |
| Birthday vs. Talked | Angry | 0.64 | .521 |
| Birthday vs. Talked | Disgusted | -0.26 | .798 |
| Birthday vs. Talked | Embarrassed | -0.72 | .475 |
| Birthday vs. Talked | Fearful | -2.36 | .019 |
| Birthday vs. Talked | Happy | -0.15 | .882 |
| Birthday vs. Talked | Hopeful | 1.09 | .278 |
| Birthday vs. Talked | Jealous | 0.99 | .325 |
| Birthday vs. Talked | Neutral | 0.00 | .998 |
| Birthday vs. Talked | Proud | 0.27 | .788 |
| Birthday vs. Talked | Sad | -4.34 | < .001 |
| Birthday vs. Talked | Sarcastic | 1.67 | .096 |
| Birthday vs. Talked | Stressed | 0.76 | .448 |
| Birthday vs. Talked | Surprised | 1.97 | .049 |

# References

Fox, J., & Weisberg, S. (2019). *An R Companion to Applied Regression* (3rd ed.). Sage. https://socialsciences.mcmaster.ca/jfox/Books/Companion/

Juslin, P. N., & Laukka, P. (2003). Communication of emotions in vocal expression and music performance: Different channels, same code? *Psychological Bulletin*, *129*(5), 770–814. https://doi.org/10.1037/0033-2909.129.5.770

Lenth, R. V. (2019). *emmeans: Estimated Marginal Means, aka Least-Squares Means*. https://cran.r-project.org/package=emmeans

Livingstone, S. R., Peck, K., & Russo, F. A. (2013). *Acoustic differences in the speaking and singing voice*. 035080–035080. https://doi.org/10.1121/1.4799460

R Core Team. (2021). *R: A language and environment for statistical computing* (4.1.2). R Foundation for Statistical Computing.

Sobin, C., & Alpert, M. (1999). Emotion in speech: The acoustic attributes of fear, anger, sadness, and joy. *Journal of Psycholinguistic Research*, *28*(4), 347–365.

Xu, Y. (2013). ProsodyPro—A tool for large-scale systematic prosody analysis. *Proceedings of Tools Resource Analysis Speech Prosody*, 7–10.
